# Surgical management of retroperitoneal cystic Schwannoma complicated by retrograde ejaculation - Case report

**DOI:** 10.1016/j.ijscr.2022.106917

**Published:** 2022-03-04

**Authors:** Saba Ilkhani, Ehsan Parvas, Sepideh Banar, Meisam Refaei, Jalalludin Khoshnevis

**Affiliations:** a Department of General and Vascular Surgery, Shohada-e-Tajrish Hospital, Shahid Beheshti University of Medical Sciences, Tehran, Iran; b School of Medicine, Shahid Beheshti University of Medical Sciences, Tehran, Iran

**Keywords:** Retroperitoneal Schwannoma, Retrograde ejaculation, Case report, Benign

## Abstract

**Introduction and importance:**

Retroperitoneal Schwannoma is unlikely to be considered in the differential diagnosis of a young patient with unexplained abdominal discomfort with no previous medical history. Tissue sampling is required for a definitive diagnosis.

**Case presentation:**

A young male presented to the emergency room with abdominal pain. Imaging study and histopathological examination confirmed the diagnosis of retroperitoneal Schwannoma. Retrograde ejaculation has been discovered as a surgical complication in follow-ups.

**Clinical discussion:**

This case is reported because of its rare clinical presentation and subsequent autonomic nerve dysfunction.

**Conclusion:**

Schwannomas are tumors of neurological origin that can grow in any location where neurons exist, and complications related to the neighboring nervous plexus should always be expected. Although uncommon, retrograde ejaculation can occur after the surgery. It is essential to inform the patient about the possibility of these complications.

## Highlights

• Retroperitoneal schwannomas are uncommon tumors with a difficult pre-operative diagnosis.
• Patients may present with nonspecific symptoms such as vague abdominal pain or genitourinary symptoms
• The treatment of choice is complete surgical excision.
• Prognosis is good but further follow up is necessary for further neurological complications

## 1 Introduction

Retroperitoneal Schwannoma is an uncommon peripheral glial cell tumor that grows slowly and is more common among female patients between 20 and 50 [1]. A rare case of this tumor was presented to the Khatam Al-Anbia specialized academic hospital in Tehran, Iran. Complete excision was performed, followed by a complication of retrograde ejaculation. The present study has been reported in line with the SCARE criteria [2].

## 2 Case presentation

**Fig. 1:**
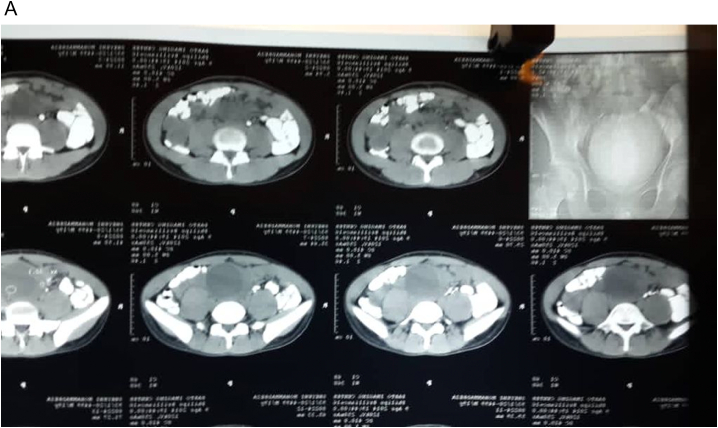

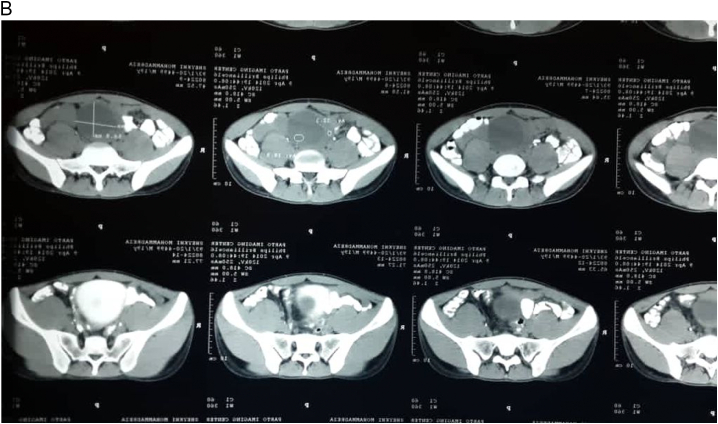
Computed tomography (CT) scan of the abdomen, Iv contrast was administered orally and intravenously. A) CT scan demonstrating a round lesion with a defined margin. B) The lesion puts pressure on the bladder's posterior left side.

A 19-year-old male with no significant medical, drug, or family history was presented to the emergency room with the chief complaint of fullness and vague abdominal pain radiating to the pelvic region since 1.5 months ago. Vital signs were stable. There were no typical neurofibromatosis type 1 clinical manifestations such as café-au-lait spots, short stature, or Lisch nodules. Laboratory studies, including complete blood count, coagulative panel, SGPT, SGOT, ALK, were within normal range. A large well-defined retroperitoneal hypodense mass was discovered on the CT scan, causing a mass effect on the bladder wall, preventing the bladder from fully being enhanced after contrast administration (Fig. 1a & b). The patient did not suffer from deep vein thrombosis or other vascular complications on the lower limb ultrasound.

**Fig. 2:**
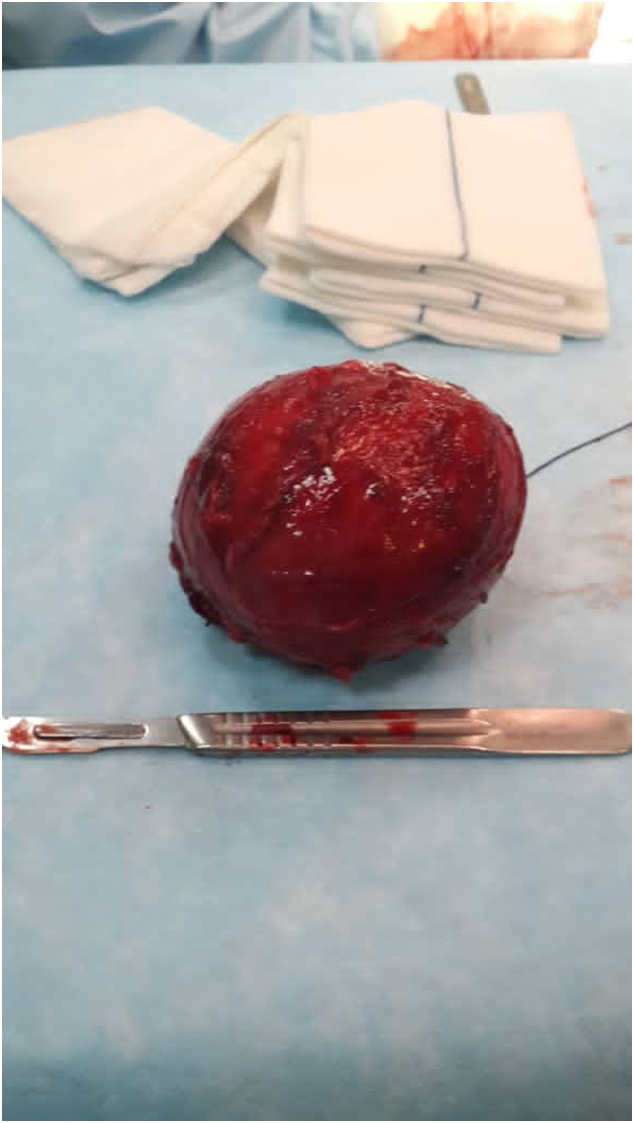
Retroperitoneal cystic Schwannoma -The well-circumscribed 8-cm-diameter tumor was excised entirely.

The patient was taken up for exploratory laparotomy. The operation was performed in a general hospital by the primary attending surgeon (with over 20 years of trainee). A well-circumstance lesion with a diameter of 8 cm was discovered anterior to aortic bifurcation and Pelvic splanchnic nerves, applying pressure on the left posterior side of the bladder, inverting into the sigmoid mesentery. Although the tumor adhered closely to the bladder and nerves (hypogastric plexus), it was encapsulated without invading the surrounding tissue allowing easy excision (Fig. 2a & b). Only one group of nerve fascicles entered the cyst, which could not be traced and isolated from the lesion. The cyst and the penetrating nerve fascicle were entirely excised.

Gross examination revealed a sharply circumscribed creamy and soft mass with a smooth external surface, measuring 8 cm in diameter. The cut section was predominantly cystic, filled by clear yellow fluid with an area of the solid component. (4 blocks).

Histopathological examination revealed a benign-appearing neoplastic lesion composed of bundles of cells having band-looking spindle nuclei without any atypical or mitotic activity. Cells bundles were arranged differently, creating palisading patterns in some regions. IHC study by S100 showed diffuse positivity. CD34, SMA-Desmin, CD117, H-caldesmon are negative. Ki-67 showed a low growth rate.

After a two-month follow-up, the patient unexpectedly complained of a sensation of ejaculation without any release of semen. Urine analysis revealed the presence of sperm. Ephedrine and pseudoephedrine were prescribed to the patient. In a three-year follow-up, the patient reported an increase in semen volume.

## 3 Discussion

Schwannomas (also known as Neurilemmomas or Neurinomas) are benign tumors of peripheral glial cells that are most typically found in head and neck regions [3]. Retroperitoneal Schwannoma is an uncommon, difficult-to-diagnose tumor that accounts for only 6% of retroperitoneal neoplasms. The paravertebral space and presacral pelvic retroperitoneum are the most common location for a retroperitoneal schwannoma [4]. Depending on their size and location, retroperitoneal schwannomas might cause a range of symptoms from asymptomatic to vague abdominal pain, bloating, and early satiety [5]. The majority of literature focuses on the diagnosis and presentation of these tumors, with only a few mentioning postoperative complications. The most commonly observed complication is recurrence (5–10%) [6]. Only two other studies [7], [8], other than ours, indicated postoperative autonomic nerve system dysfunction due to Nervi Erigentes' manipulation. The Nervi Erigentes are splanchnic nerves that arise from sacral spinal nerves that migrate through the hypogastric plexus along the cavernous nerves, providing parasympathetic innervation to erectile bodies [9]. The most probable explanation for following retrograde ejaculation reported in our patients and those two other patients discussed in publications is an injury to these regions during operation. Although we employed open surgery in our study, two other studies used laparoscopic surgery. It can be interpreted that the type of procedure is not the cause of the complication, and the neural nature of the Schwannoma is the most likely reason for this neurological complication. According to a new study, utilizing a virtual-navigation strategy is beneficial. Preoperative simulation and intraoperative navigation utilizing 3D images are effective methods for increasing a surgeon's understanding of a patient's anatomy and are particularly useful for resecting a retroperitoneal tumor at a deep and difficult anatomical region [10]. As previously stated, only one nerve fascicle group entered the lesion of this case, and the cyst was completely excised due to its large diameter and malignant potential. We did not expect a single nerve fiber deficit in the field to result in retrograde ejaculaton.

In Conclusion, Retroperitoneal schwannomas are challenging to diagnose before surgery and require pathologic examination to confirm the diagnosis. They are prone to misdiagnose as malignant tumors [11], [12]. The surgeon should expect neurologic complications based on the proximity to the neighboring neural plexus. Regardless of the rarity, It is critical to inform the patient of the possibility of neurological side effects. Retrograde ejaculation can result from damage to Nerve Ergentis, and it is necessary to ensure that this complication will not affect the patients fertility.

The patient had a positive perspective toward his surgery at the time of discharge, and he only experienced bearable pain at the surgical site. However, in follow-up sessions, The patient became dissatisfied with the side effect of retrograde ejaculation and was concerned about his sterility. After undergoing medical therapy, the patient stated that his condition had improved.

## Provenance and peer review

Not commissioned, externally peer-reviewed.

## Source of funding

The authors received no specific funding for this work.

## Ethical approval

The ethical committee of Shahid Beheshti University approved the case-report.

## Consent

Written informed consent was obtained from the patient to publish this case report and accompanying images. A copy of the written consent is available for review by the Editor-in-Chief of this journal on request.

## Research registration

(for case reports detailing a new surgical technique or new equipment/technology): N/A.

## Guarantor

Jalaluddin Khoshnevis is the Guarantor of this study.

## CRediT authorship contribution statement

SI, SB, MR, EP, and JK were involved in the study concept or design.

MR, EP, and JK were involved in data collection.

SI and SB were involved in data interpretation.

SI, EP, MR were involved in writing the paper under the supervision of JK.

## Declaration of competing interest

The authors declare that there are no conflicts of interests.
